# Crystal structure of allyl­ammonium hydrogen succinate at 100 K

**DOI:** 10.1107/S1600536814015633

**Published:** 2014-08-01

**Authors:** Błażej Dziuk, Bartosz Zarychta, Krzysztof Ejsmont

**Affiliations:** a Faculty of Chemistry, University of Opole, Oleska 48, 45-052 Opole, Poland

**Keywords:** crystal structure, allyl­ammonium, succinate, hydrogen bonds

## Abstract

The asymmetric unit of the title compound, C_2_H_8_N^+^·C_4_H_5_O_4_^−^, consists of two allyl­ammonium cations and two hydrogen succinate anions (*Z*′ = 2). One of the cations has a near-perfect *syn*-periplanar (*cis*) conformation with an N—C—C—C torsion angle of 0.4 (3)°, while the other is characterized by a *gauche* conformation and a torsion angle of 102.5 (3)°. Regarding the anions, three out of four carboxilic groups are twisted with respect to the central C–CH_2_–CH_2_–C group [dihedral angles = 24.4 (2), 31.2 (2) and 40.4 (2)°], the remaining one being instead almost coplanar, with a dihedral angle of 4.0 (2)°. In the crystal, there are two very short, near linear O—H⋯O hydrogen bonds between anions, with the H atoms shifted notably from the donor O towards the O⋯O midpoint. These O—H⋯O hydrogen bonds form helical chains along the [011] which are further linked to each other through N—H⋯O hydrogen bonds (involving all the available NH groups), forming layers lying parallel to (100).

## Related literature   

For other crystal structures of succinate salts with amines, see: Bhardwaj *et al.* (2013 ▶); Bruni *et al.* (2013 ▶); Khorasani & Fernandes (2012 ▶). For the characteristic structural motifs in ammonium di­carboxyl­ate salts, see: Kashino *et al.* (1998 ▶); Barnes & Weakley (2000 ▶); MacDonald *et al.* (2001 ▶); Vaidhyanathan *et al.* (2001 ▶, 2002 ▶); Saraswathi & Vijayan (2002 ▶); Ejsmont (2007 ▶). Salts of succinic acid and amines have strong N—H⋯O and O—H⋯O hydrogen bonds and are thus used as building blocks for the construction of supra­molecular structures, see: Khorasani *et al.* (2012 ▶); Lemmerer (2011 ▶). For hydrogen bonding, see: Steiner (2002 ▶). For a description of the Cambridge Structural Database, see: Allen (2002 ▶).
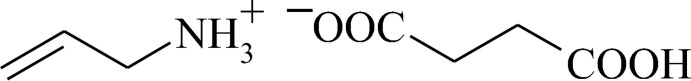


## Experimental   

### Crystal data  

C_3_H_8_N^+^·C_4_H_5_O_4_^−^
*M* *_r_* = 175.19
Triclinic, 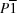

*a* = 8.5649 (3) Å
*b* = 9.4364 (3) Å
*c* = 10.8051 (4) Å
α = 88.838 (3)°
β = 87.482 (3)°
γ = 82.843 (3)°
*V* = 865.55 (5) Å^3^
*Z* = 4
Mo *K*α radiation
μ = 0.11 mm^−1^
*T* = 100 K
0.30 × 0.20 × 0.15 mm

### Data collection  

Oxford Diffraction Xcalibur diffractometer
5454 measured reflections
3013 independent reflections
2373 reflections with *I* > 2σ*I*)
*R*_int_ = 0.014

### Refinement  

*R*[*F* ^2^ > 2σ(*F* ^2^)] = 0.036
*wR*(*F* ^2^) = 0.093
*S* = 1.10
3013 reflections
249 parameters
H atoms treated by a mixture of independent and constrained refinement
Δρ_max_ = 0.57 e Å^−3^
Δρ_min_ = −0.50 e Å^−3^

### 

Data collection: *CrysAlis CCD* (Oxford Diffraction, 2008 ▶); cell refinement: *CrysAlis RED* (Oxford Diffraction, 2008 ▶); data reduction: *CrysAlis RED*; program(s) used to solve structure: *SHELXS97* (Sheldrick, 2008 ▶); program(s) used to refine structure: *SHELXL97* (Sheldrick, 2008 ▶); molecular graphics: *SHELXTL* (Sheldrick, 2008 ▶); software used to prepare material for publication: *SHELXL97*.

## Supplementary Material

Crystal structure: contains datablock(s) global, I. DOI: 10.1107/S1600536814015633/bg2532sup1.cif

Structure factors: contains datablock(s) I. DOI: 10.1107/S1600536814015633/bg2532Isup2.hkl

Supporting information file. DOI: 10.1107/S1600536814015633/bg2532Isup3.cml

**Fig. 1.**
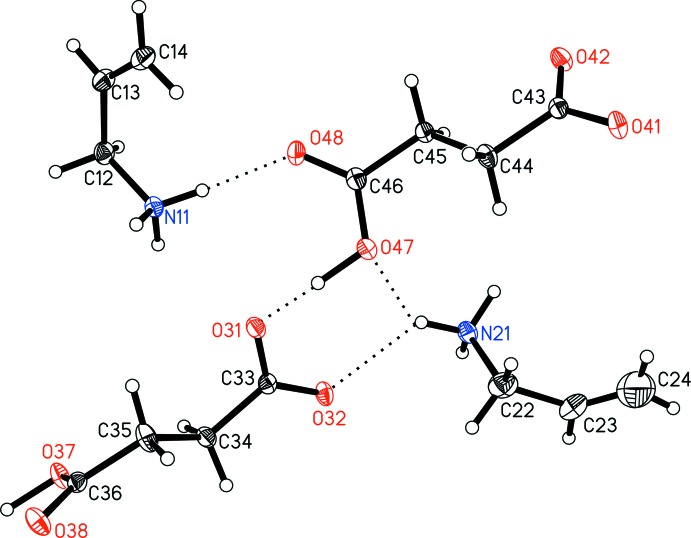
The molecular structure of (I), showing 50% displacement ellipsoids. Hydrogen bonds are shown as dotted lines.

**Fig. 2.**
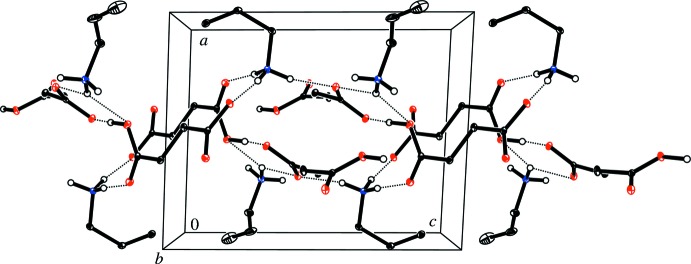
Packing diagram of (I) viewed along the b axis, showing (sideways) the (100) 2D structure defined by the hydrogen-bonding network (dotted lines).

. DOI: 10.1107/S1600536814015633/bg2532fig1.tif
The mol­ecular structure of (I), showing 50% displacement ellipsoids. Hydrogen bonds are shown as dotted lines.

b . DOI: 10.1107/S1600536814015633/bg2532fig2.tif
Packing diagram of (I) viewed along the *b* axis, showing (sideways) the (100) 2D structure defined by the hydrogen-bonding network (dotted lines).

CCDC reference: 1012134

Additional supporting information:  crystallographic information; 3D view; checkCIF report

## Figures and Tables

**Table 1:** Hydrogen-bond geometry (Å, °)

*D*—H⋯*A*	*D*—H	H⋯*A*	*D*⋯*A*	*D*—H⋯*A*
N11—H11*A*⋯O48	0.94 (2)	1.89 (2)	2.8275 (19)	174.4 (17)
N11—H11*B*⋯O32^i^	0.95 (2)	1.88 (2)	2.8107 (19)	166.9 (18)
N11—H11*C*⋯O41^ii^	0.90 (2)	1.95 (2)	2.844 (2)	172 (2)
N21—H21*A*⋯O32	0.95 (3)	2.28 (3)	2.972 (2)	128.5 (19)
N21—H21*A*⋯O47	0.95 (3)	2.21 (3)	2.994 (2)	138.7 (19)
N21—H21*B*⋯O42^iii^	0.93 (2)	1.86 (2)	2.786 (2)	169.2 (17)
N21—H21*C*⋯O38^iv^	0.92 (2)	1.86 (2)	2.7809 (19)	177 (2)
O37—H37⋯O41^v^	1.18 (3)	1.28 (3)	2.4510 (15)	180 (3)
O47—H47⋯O31	1.08 (3)	1.39 (3)	2.4707 (15)	176 (3)

Symmetry codes: (i) 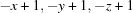; (ii) 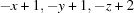; (iii) 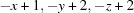; (iv) 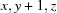; (v) 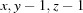.

## supplementary crystallographic information

### S1. Comment

Crystal engineering is extremely fast growing area of experimental chemistry leading to new materials with controlled and understood nature. Hydrogen bonding plays an important role in organizing molecules, assembling them to create supramolecules and controlling their dimensions in one-, two- or three-dimensions (Khorasani *et al.*, 2012). The adducts of succinic acid and amines have strong N—H···O and O—H···O hydrogen bonds, thus they can be used to align molecules in chosen directions, as building blocks for the construction of supramolecular structures. (Khorasani *et al.*, 2012; Lemmerer, 2011).

There are three characteristic structural motifs in ammonium dicarboxylate salts: (i) linear chains of dicarboxylic acids formed by strong hydrogen bonds; (ii) dimers of dicarboxylic acid molecules; (iii) isolated oxalate monoanions or dianion units (for example: Kashino *et al.*, 1998; Barnes & Weakley 2000; MacDonald *et al.*, 2001; Vaidhyanathan *et al.*, 2001, 2002; Saraswathi & Vijayan 2002; and Ejsmont, 2007).

The independent part of the unit cell of the title salt, (I), consists with two allyloammonium cations and two hydrogen succinate anions (Fig. 1). A geometry of amonium cations is normal (CSD; CONQUEST Version 1.16; Allen, 2002) and comparable with those found in other crystal structures which include this cation (Allen, 2002). The N11 cation has perfect *syn*-periplanar (*cis*) conformation with N11–C12–C13–C14 torsion angle of 0.4 (3)°, while N21 cataion is characterized by *gauche* conformation (the torsion angle N21–C22–C23—C24 amounts 102.5 (3)°). Three out of four carboxalic groups are twisted with respect to the central C–CH_2_–CH_2_–C group; the remaining one being rather co-planar.

In the crystal structure of (I), there are two linear or nearly linear O–H···O hydrogen bonds between the hydrogen succinate, which can be identified as a very strong interactions (Steiner, 2002). The O···O distances in these interactions are close to that observed for O–H···O hydrogen bonds formed between the monoanionic oxalate units in the structures of diethylammonium hydrogen oxalate (Ejsmont, 2007). These O–H···O hydrogen bonds forming helical chains along <011> direction. The allylammonium cations are linked to polianionic chains through the N–H···O hydrogen bonds (Table 2, Fig. 2).

### S2. Experimental

Crystals of (I) were grown at room temperature by slow evaporation of an aqueous solution containing allylamine and succinatic acid in a 1:1 stoichiometric ratio.

### S3. Refinement

The H atoms attached to atoms O and N were located in difference electron density maps and were freely refined with isotropic displacement factors [O–H = 1.08 (3) - 1.18 (3) and N–H = 0.90 (2) - 0.95 (2) Å]. The remaining H atoms were positioned geometrically and treated as riding on their parent C atoms, with C–H distances of 0.95 for idealized secondary CH_2_, 0.95 for CH and 0.99 Å for idealized terminal *X*=CH_2_ and with *U*_iso_(H) = 1.2*U*_eq_(C). Probably due to libration, the ending C23═C24 bond appears significantly shorter that its corresponding C13═C14 one.

### Crystal data

**Table:**

C_3_H_8_N^+^·C_4_H_5_O_4_^−^	*Z* = 4
*M**_r_* = 175.19	*F*(000) = 376
Triclinic, *P*1	*D*_x_ = 1.344 Mg m^−^^3^
Hall symbol: -P 1	Mo *K*α radiation, λ = 0.71073 Å
*a* = 8.5649 (3) Å	Cell parameters from 5943 reflections
*b* = 9.4364 (3) Å	θ = 2.9–26.0°
*c* = 10.8051 (4) Å	µ = 0.11 mm^−^^1^
α = 88.838 (3)°	*T* = 100 K
β = 87.482 (3)°	Prism, colourless
γ = 82.843 (3)°	0.30 × 0.20 × 0.15 mm
*V* = 865.55 (5) Å^3^	

### Data collection

**Table:**

Oxford Diffraction Xcalibur diffractometer	2373 reflections with *I* > 2σ*I*)
Radiation source: fine-focus sealed tube	*R*_int_ = 0.014
Graphite monochromator	θ_max_ = 25.0°, θ_min_ = 2.9°
ω–scan	*h* = −10→10
5454 measured reflections	*k* = −11→11
3013 independent reflections	*l* = −8→12

### Refinement

**Table:**

Refinement on *F*^2^	Primary atom site location: structure-invariant direct methods
Least-squares matrix: full	Secondary atom site location: difference Fourier map
*R*[*F*^2^ > 2σ(*F*^2^)] = 0.036	Hydrogen site location: inferred from neighbouring sites
*wR*(*F*^2^) = 0.093	H atoms treated by a mixture of independent and constrained refinement
*S* = 1.10	*w* = 1/[σ^2^(*F*_o_^2^) + (0.0483*P*)^2^ + 0.1223*P*] where *P* = (*F*_o_^2^ + 2*F*_c_^2^)/3
3013 reflections	(Δ/σ)_max_ < 0.001
249 parameters	Δρ_max_ = 0.57 e Å^−^^3^
0 restraints	Δρ_min_ = −0.50 e Å^−^^3^

### Special details

**Table:**

Geometry. All e.s.d.'s (except the e.s.d. in the dihedral angle between two l.s. planes) are estimated using the full covariance matrix. The cell e.s.d.'s are taken into account individually in the estimation of e.s.d.'s in distances, angles and torsion angles; correlations between e.s.d.'s in cell parameters are only used when they are defined by crystal symmetry. An approximate (isotropic) treatment of cell e.s.d.'s is used for estimating e.s.d.'s involving l.s. planes.
Refinement. Refinement of *F*^2^ against ALL reflections. The weighted *R*-factor *wR* and goodness of fit *S* are based on *F*^2^, conventional *R*-factors *R* are based on *F*, with *F* set to zero for negative *F*^2^. The threshold expression of *F*^2^ > σ(*F*^2^) is used only for calculating *R*-factors(gt) *etc*. and is not relevant to the choice of reflections for refinement. *R*-factors based on *F*^2^ are statistically about twice as large as those based on *F*, and *R*- factors based on ALL data will be even larger.

### Fractional atomic coordinates and isotropic or equivalent isotropic displacement parameters (Å^2^)

**Table:**

	*x*	*y*	*z*	*U*_iso_*/*U*_eq_	
N11	0.22162 (17)	0.36564 (17)	0.68154 (14)	0.0143 (3)	
H11A	0.240 (2)	0.444 (2)	0.7297 (18)	0.022 (5)*	
H11B	0.263 (2)	0.387 (2)	0.601 (2)	0.026 (5)*	
H11C	0.275 (3)	0.284 (2)	0.711 (2)	0.035 (6)*	
C12	0.0522 (2)	0.34987 (19)	0.67065 (15)	0.0171 (4)	
H12A	0.0440	0.2616	0.6284	0.021*	
H12B	0.0041	0.4279	0.6199	0.021*	
C13	−0.0375 (2)	0.34861 (19)	0.79193 (16)	0.0195 (4)	
H13	−0.1441	0.3396	0.7890	0.023*	
C14	0.0178 (2)	0.35885 (19)	0.90250 (16)	0.0213 (4)	
H14A	0.1236	0.3681	0.9107	0.026*	
H14B	−0.0491	0.3569	0.9726	0.026*	
N21	0.72616 (18)	0.81019 (17)	0.70187 (15)	0.0163 (3)	
H21A	0.670 (3)	0.729 (3)	0.699 (2)	0.053 (7)*	
H21B	0.675 (2)	0.880 (2)	0.7549 (18)	0.023 (5)*	
H21C	0.734 (3)	0.847 (2)	0.623 (2)	0.037 (6)*	
C22	0.8872 (2)	0.7596 (2)	0.74249 (19)	0.0266 (5)	
H22A	0.9356	0.6839	0.6888	0.032*	
H22B	0.8813	0.7213	0.8263	0.032*	
C23	0.9854 (3)	0.8799 (3)	0.7381 (3)	0.0480 (7)	
H23	1.0230	0.9037	0.6593	0.058*	
C24	1.0244 (3)	0.9520 (3)	0.8213 (3)	0.0649 (9)	
H24A	0.9915	0.9347	0.9028	0.078*	
H24B	1.0869	1.0241	0.8033	0.078*	
O31	0.56273 (14)	0.40484 (12)	0.67627 (10)	0.0164 (3)	
O32	0.70239 (14)	0.54666 (12)	0.56148 (10)	0.0167 (3)	
C33	0.64907 (19)	0.43020 (17)	0.58151 (15)	0.0129 (4)	
C34	0.6844 (2)	0.31171 (17)	0.48796 (15)	0.0156 (4)	
H34A	0.6139	0.3315	0.4202	0.019*	
H34B	0.7911	0.3127	0.4543	0.019*	
C35	0.6679 (2)	0.16362 (18)	0.53950 (16)	0.0195 (4)	
H35A	0.7512	0.1369	0.5966	0.023*	
H35B	0.5683	0.1670	0.5864	0.023*	
C36	0.6745 (2)	0.04877 (18)	0.44309 (15)	0.0142 (4)	
O37	0.60445 (14)	0.08573 (12)	0.34227 (10)	0.0178 (3)	
H37	0.614 (3)	−0.012 (3)	0.275 (2)	0.060 (7)*	
O38	0.73921 (15)	−0.07378 (12)	0.46355 (11)	0.0199 (3)	
O41	0.62286 (14)	0.88262 (12)	1.20228 (10)	0.0168 (3)	
O42	0.39008 (14)	0.97603 (12)	1.13231 (10)	0.0177 (3)	
C43	0.5051 (2)	0.88300 (18)	1.13021 (14)	0.0135 (4)	
C44	0.5199 (2)	0.76104 (17)	1.04077 (15)	0.0153 (4)	
H44A	0.5367	0.6717	1.0871	0.018*	
H44B	0.6118	0.7668	0.9860	0.018*	
C45	0.3767 (2)	0.75980 (18)	0.96308 (15)	0.0159 (4)	
H45A	0.2854	0.7521	1.0181	0.019*	
H45B	0.3586	0.8503	0.9186	0.019*	
C46	0.3904 (2)	0.64079 (18)	0.87107 (15)	0.0145 (4)	
O47	0.53341 (14)	0.60086 (13)	0.82691 (11)	0.0182 (3)	
H47	0.543 (3)	0.518 (3)	0.758 (3)	0.084 (10)*	
O48	0.27396 (14)	0.58861 (13)	0.83964 (11)	0.0193 (3)	

### Atomic displacement parameters (Å^2^)

**Table:**

	*U*^11^	*U*^22^	*U*^33^	*U*^12^	*U*^13^	*U*^23^
N11	0.0163 (8)	0.0133 (8)	0.0138 (8)	−0.0028 (6)	−0.0011 (6)	−0.0018 (6)
C12	0.0158 (9)	0.0179 (9)	0.0181 (9)	−0.0025 (7)	−0.0045 (7)	−0.0022 (7)
C13	0.0149 (9)	0.0204 (10)	0.0235 (10)	−0.0042 (7)	0.0008 (8)	−0.0010 (8)
C14	0.0201 (10)	0.0238 (10)	0.0211 (10)	−0.0081 (8)	0.0034 (8)	0.0000 (8)
N21	0.0194 (8)	0.0115 (8)	0.0177 (8)	−0.0008 (7)	−0.0016 (7)	−0.0008 (7)
C22	0.0207 (10)	0.0257 (11)	0.0321 (11)	0.0027 (8)	−0.0028 (8)	0.0018 (9)
C23	0.0229 (12)	0.0636 (17)	0.0608 (16)	−0.0184 (12)	0.0116 (11)	−0.0269 (14)
C24	0.0543 (18)	0.0384 (16)	0.104 (2)	−0.0198 (13)	0.0227 (17)	−0.0188 (16)
O31	0.0208 (7)	0.0143 (6)	0.0147 (6)	−0.0052 (5)	0.0034 (5)	−0.0038 (5)
O32	0.0240 (7)	0.0115 (6)	0.0155 (6)	−0.0061 (5)	−0.0002 (5)	−0.0009 (5)
C33	0.0129 (8)	0.0136 (9)	0.0125 (8)	−0.0008 (7)	−0.0043 (7)	−0.0006 (7)
C34	0.0190 (9)	0.0133 (9)	0.0146 (9)	−0.0026 (7)	0.0022 (7)	−0.0022 (7)
C35	0.0311 (11)	0.0140 (9)	0.0136 (9)	−0.0027 (8)	−0.0036 (8)	−0.0015 (7)
C36	0.0150 (9)	0.0144 (9)	0.0137 (9)	−0.0042 (7)	0.0015 (7)	−0.0004 (7)
O37	0.0251 (7)	0.0123 (6)	0.0161 (6)	0.0000 (5)	−0.0060 (5)	−0.0037 (5)
O38	0.0294 (7)	0.0124 (6)	0.0169 (6)	0.0024 (5)	−0.0022 (5)	−0.0007 (5)
O41	0.0213 (7)	0.0137 (6)	0.0158 (6)	−0.0016 (5)	−0.0057 (5)	−0.0031 (5)
O42	0.0197 (7)	0.0157 (6)	0.0174 (6)	0.0010 (5)	−0.0019 (5)	−0.0045 (5)
C43	0.0186 (9)	0.0118 (8)	0.0109 (8)	−0.0061 (7)	0.0017 (7)	0.0009 (7)
C44	0.0215 (9)	0.0108 (8)	0.0136 (8)	−0.0021 (7)	−0.0013 (7)	−0.0007 (7)
C45	0.0177 (9)	0.0162 (9)	0.0147 (9)	−0.0053 (7)	0.0015 (7)	−0.0037 (7)
C46	0.0199 (9)	0.0123 (9)	0.0118 (8)	−0.0041 (7)	−0.0002 (7)	0.0020 (7)
O47	0.0195 (7)	0.0168 (7)	0.0188 (6)	−0.0047 (5)	0.0026 (5)	−0.0060 (5)
O48	0.0207 (7)	0.0194 (7)	0.0196 (6)	−0.0087 (5)	−0.0007 (5)	−0.0051 (5)

### Geometric parameters (Å, º)

**Table:**

N11—C12	1.487 (2)	O32—C33	1.253 (2)
N11—H11A	0.94 (2)	C33—C34	1.516 (2)
N11—H11B	0.95 (2)	C34—C35	1.515 (2)
N11—H11C	0.90 (2)	C34—H34A	0.9700
C12—C13	1.490 (2)	C34—H34B	0.9700
C12—H12A	0.9700	C35—C36	1.513 (2)
C12—H12B	0.9700	C35—H35A	0.9700
C13—C14	1.314 (2)	C35—H35B	0.9700
C13—H13	0.9300	C36—O38	1.239 (2)
C14—H14A	0.9300	C36—O37	1.288 (2)
C14—H14B	0.9300	O37—H37	1.18 (3)
N21—C22	1.484 (2)	O41—C43	1.301 (2)
N21—H21A	0.95 (3)	O42—C43	1.235 (2)
N21—H21B	0.93 (2)	C43—C44	1.508 (2)
N21—H21C	0.92 (2)	C44—C45	1.518 (2)
C22—C23	1.494 (3)	C44—H44A	0.9700
C22—H22A	0.9700	C44—H44B	0.9700
C22—H22B	0.9700	C45—C46	1.505 (2)
C23—C24	1.220 (4)	C45—H45A	0.9700
C23—H23	0.9300	C45—H45B	0.9700
C24—H24A	0.9300	C46—O48	1.230 (2)
C24—H24B	0.9300	C46—O47	1.308 (2)
O31—C33	1.273 (2)	O47—H47	1.08 (3)
O31—H47	1.39 (3)		
			
C12—N11—H11A	113.9 (12)	O32—C33—C34	119.48 (14)
C12—N11—H11B	107.5 (12)	O31—C33—C34	116.77 (14)
H11A—N11—H11B	104.3 (16)	C35—C34—C33	114.54 (14)
C12—N11—H11C	110.7 (14)	C35—C34—H34A	108.6
H11A—N11—H11C	110.4 (17)	C33—C34—H34A	108.6
H11B—N11—H11C	109.9 (18)	C35—C34—H34B	108.6
N11—C12—C13	113.82 (14)	C33—C34—H34B	108.6
N11—C12—H12A	108.8	H34A—C34—H34B	107.6
C13—C12—H12A	108.8	C36—C35—C34	114.80 (14)
N11—C12—H12B	108.8	C36—C35—H35A	108.6
C13—C12—H12B	108.8	C34—C35—H35A	108.6
H12A—C12—H12B	107.7	C36—C35—H35B	108.6
C14—C13—C12	127.08 (17)	C34—C35—H35B	108.6
C14—C13—H13	116.5	H35A—C35—H35B	107.5
C12—C13—H13	116.5	O38—C36—O37	123.12 (15)
C13—C14—H14A	120.0	O38—C36—C35	121.04 (15)
C13—C14—H14B	120.0	O37—C36—C35	115.80 (15)
H14A—C14—H14B	120.0	C36—O37—H37	110.1 (12)
C22—N21—H21A	108.0 (14)	O42—C43—O41	123.53 (15)
C22—N21—H21B	111.4 (12)	O42—C43—C44	121.67 (15)
H21A—N21—H21B	111.1 (19)	O41—C43—C44	114.79 (14)
C22—N21—H21C	108.6 (14)	C43—C44—C45	113.51 (14)
H21A—N21—H21C	108 (2)	C43—C44—H44A	108.9
H21B—N21—H21C	110.0 (18)	C45—C44—H44A	108.9
N21—C22—C23	110.26 (17)	C43—C44—H44B	108.9
N21—C22—H22A	109.6	C45—C44—H44B	108.9
C23—C22—H22A	109.6	H44A—C44—H44B	107.7
N21—C22—H22B	109.6	C46—C45—C44	114.39 (14)
C23—C22—H22B	109.6	C46—C45—H45A	108.7
H22A—C22—H22B	108.1	C44—C45—H45A	108.7
C24—C23—C22	130.4 (3)	C46—C45—H45B	108.7
C24—C23—H23	114.8	C44—C45—H45B	108.7
C22—C23—H23	114.8	H45A—C45—H45B	107.6
C23—C24—H24A	120.0	O48—C46—O47	123.71 (15)
C23—C24—H24B	120.0	O48—C46—C45	121.43 (15)
H24A—C24—H24B	120.0	O47—C46—C45	114.85 (15)
C33—O31—H47	112.0 (12)	C46—O47—H47	115.2 (16)
O32—C33—O31	123.73 (15)		

### Hydrogen-bond geometry (Å, º)

**Table:**

*D*—H···*A*	*D*—H	H···*A*	*D*···*A*	*D*—H···*A*
N11—H11*A*···O48	0.94 (2)	1.89 (2)	2.8275 (19)	174.4 (17)
N11—H11*B*···O32^i^	0.95 (2)	1.88 (2)	2.8107 (19)	166.9 (18)
N11—H11*C*···O41^ii^	0.90 (2)	1.95 (2)	2.844 (2)	172 (2)
N21—H21*A*···O32	0.95 (3)	2.28 (3)	2.972 (2)	128.5 (19)
N21—H21*A*···O47	0.95 (3)	2.21 (3)	2.994 (2)	138.7 (19)
N21—H21*B*···O42^iii^	0.93 (2)	1.86 (2)	2.786 (2)	169.2 (17)
N21—H21*C*···O38^iv^	0.92 (2)	1.86 (2)	2.7809 (19)	177 (2)
O37—H37···O41^v^	1.18 (3)	1.28 (3)	2.4510 (15)	180 (3)
O47—H47···O31	1.08 (3)	1.39 (3)	2.4707 (15)	176 (3)

Symmetry codes: (i) −*x*+1, −*y*+1, −*z*+1; (ii) −*x*+1, −*y*+1, −*z*+2; (iii) −*x*+1, −*y*+2, −*z*+2; (iv) *x*, *y*+1, *z*; (v) *x*, *y*−1, *z*−1.
