# Supplementary figures and images for: Crystal structure of allyl­ammonium hydrogen succinate at 100 K
Source: Acta Crystallogr Sect E Struct Rep Online. 2014 Aug 1;70(Pt 9):o917–8. doi: 10.1107/S1600536814015633 (PMC4186065; doi:10.1107/S1600536814015633)

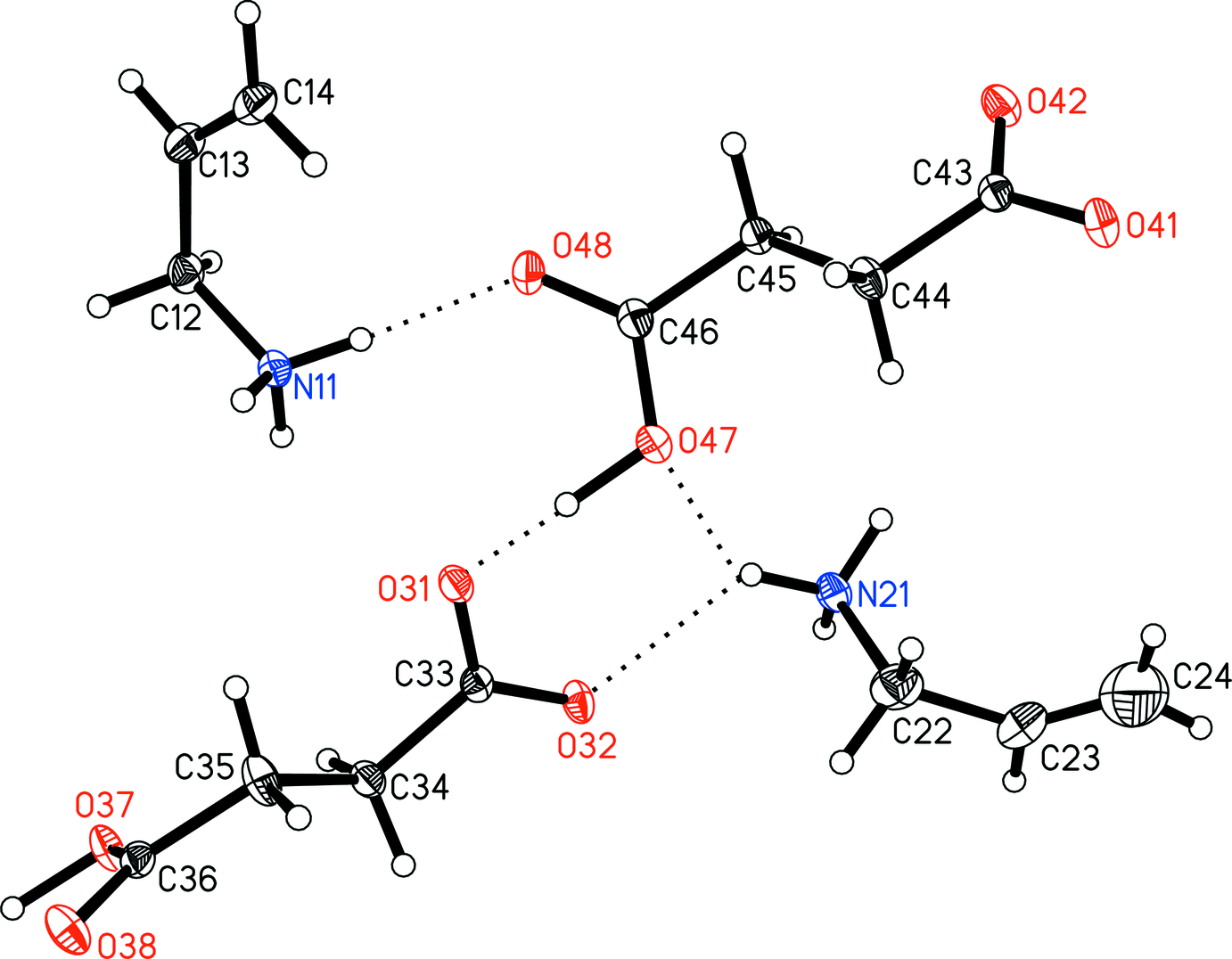

Supplement: Supplementary file 4 [file e-70-0o917-fig1.tif]

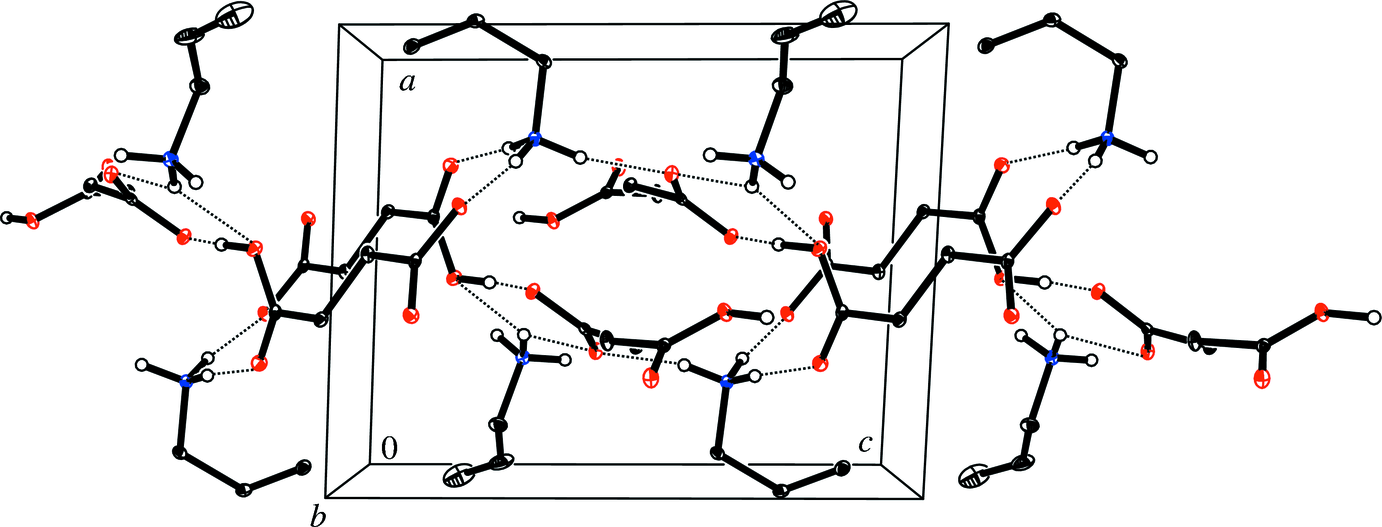

Supplement: Supplementary file 5 [file e-70-0o917-fig2.tif]
